# The Influence of Plasma Treatment on the Corrosion and Biocompatibility of Magnesium

**DOI:** 10.3390/ma15207405

**Published:** 2022-10-21

**Authors:** Aleksandra Kocijan, Janez Kovač, Ita Junkar, Matic Resnik, Veno Kononenko, Marjetka Conradi

**Affiliations:** 1 Institute of Metals and Technology, Lepi Pot 11, 1000 Ljubljana, Slovenia; 2 Jozef Stefan Institute, Jamova 39, 1000 Ljubljana, Slovenia; 3 Biotechnical Faculty, University of Ljubljana, Večna Pot 111, 1000 Ljubljana, Slovenia

**Keywords:** magnesium, plasma treatment, surface chemistry, topography, wetting, corrosion, biocompatibility

## Abstract

In our study, plasma surface modification was employed to tailor the surface properties of magnesium in terms of surface chemistry, topography, and wettability. For two sets of samples, the plasma treatment involved two steps using two different gases (hydrogen and oxygen), while one set of samples was treated with one step only using oxygen. X-ray photoelectron spectroscopy (XPS) was applied to determine the surface composition, oxidation state of the elements, and the thickness of the surface oxide layer on the Mg samples after different plasma treatments. The surface morphology was characterised using atomic force microscopy (AFM) and scanning electron microscopy (SEM). The wettability was analysed by measuring the static water-contact angles and the corrosion was evaluated using potentiodynamic measurements. The interaction of the live cells with the differently modified Mg surfaces was evaluated in terms of biocompatibility using MG-63 cells (human bone osteosarcoma cells). We have shown that a plasma surface treatment significantly decreased the carbon content and the formation of a 15–20-nm-thick MgO layer was observed. This improves the corrosion resistance, while the biocompatibility was retained, compared to the untreated Mg. A plasma surface treatment is therefore an important step in the development of novel surfaces with improved corrosion resistance for magnesium in biomedical applications.

## 1. Introduction

Over the past few years, magnesium and its alloys have attracted increasing attention as promising biodegradable metallic materials. They show superior biocompatibility [1,2,3] and biodegradability [4,5,6], they have a low density, and they have mechanical properties most similar to bone, in comparison with other metallic materials [4]. These advantages enable magnesium and its alloys to serve as temporary implants, avoiding the problems associated with permanent metallic implants in terms of stress shielding [7], inflammation [8], interference in radiological investigations [9], and subsequent surgeries for implant removal that result in health-related issues and additional costs.

Magnesium is considered to be non-toxic because it is one of the most essential elements in the human body. However, it corrodes rapidly under physiological conditions, which leads to local hydrogen evolution and alkalisation. An oxide layer, predominantly consisting of Mg(OH)_2_, is porous and exhibits poor barrier properties, leading to severe corrosion propagation [10]. The porous corrosion layer is therefore considered to be one of the main reasons for the poor corrosion properties of Mg alloys [10,11]. The high biodegradability reduces the mechanical stability before achieving sufficient tissue recovery [12]. To overcome these drawbacks, different approaches have been proposed to improve the mechanical and corrosion properties: alloying with different elements, microstructure tailoring and surface modification with either physico-chemical approaches or coatings [13,14,15,16,17,18,19,20,21]. Plasma surface modification is a well-known tool for tailoring the surface properties without interfering with the properties of the bulk material. Various approaches using plasma for the modification of magnesium to slow down its degradation rate have already been proposed: plasma electrolytic oxidation [22], plasma immersion ion implantation and deposition [23], plasma spraying [24], and laser plasma technologies [25]. Plasma electrolytic oxidation (PEO) is frequently used, as it allows the simple formation of an oxide layer on the surface of a magnesium alloy [12]. In this case the plasma is generated by applying an extremely high voltage in a suitable electrolyte. This study focuses on the plasma surface modification of magnesium, where a direct exposure to gaseous, low-pressure, inductively coupled radio-frequency plasma was used to improve its biocompatibility and corrosion properties. To the best of our knowledge, this is the first report on such a treatment.

The main emphasis is to study the effects of different plasma surface modifications of magnesium to tailor the surface properties in terms of surface chemistry, topography, and wettability, which in turn significantly influences the corrosion resistance and biocompatibility of the surface. The aim of the direct plasma treatment was to artificially create a new, thicker, and pinhole-free oxide layer on the surface of magnesium samples. Different treatment plasma conditions were studied; in two cases, hydrogen plasma was first used for the reduction of the native magnesium oxide layer (step 1) followed by the oxygen plasma (step 2) to enable the formation of a new, improved oxide layer. In one case, only the oxygen plasma was tested (step 2) to observe whether it can improve the native oxide layer without the need to use hydrogen. The surface characterisation of the plasma-modified samples was analysed by X-ray photoelectron spectroscopy (XPS), atomic force microscopy (AFM), and scanning electron microscopy (SEM). Their wettability was revealed by measuring static water-contact angles, while corrosion was evaluated by using potentiodynamic measurements. The interactions of live cells with differently modified Mg surfaces were evaluated using MG-63 cells (human bone osteosarcoma cells) that behave in a similar way to osteoblasts and are commonly used for the evaluation of biocompatibility and cellular responses to different materials [26]. The plasma-surface-modification approach represents an important step in the development of novel surfaces for the improved corrosion resistance of magnesium in clinical use as biodegradable implants and cardiovascular applications [27].

## 2. Materials and Methods

*Materials*. A magnesium rod (Goodfellow, 25.4 mm in diameter, 99.9% purity) was cut into discs, ground with SiC emery paper up to 4000 grit, diamond polished up to 1 µm, ultrasonically cleaned with ethanol and dried in warm air prior to plasma surface modification.

**Figure 1:**
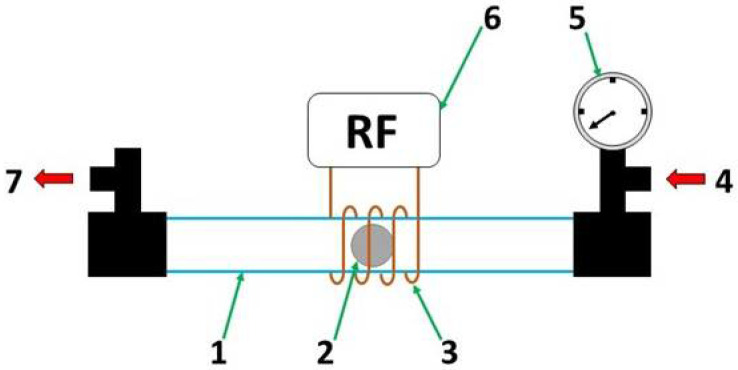
Plasma treatment system: (1) quartz tube, (2) magnesium disc—sample, (3) copper coil, (4) gas inlet, (5) pressure gauge, (6) radio-frequency generator at 13.56 MHz with matching network, (7) vacuum pump.

*Plasma surface modification*. Plasma surface modifications were performed using a low-pressure inductively coupled radio-frequency plasma system. The system includes a 70-cm-long glass tube with a 3.5 cm internal diameter. The gas inlet and the pressure gauge were mounted on one side of the tube and the vacuum pump on the other. In the middle there was a copper coil wrapped around the tube, as shown in Figure 1. After inserting the polished magnesium disc placed on a quartz object slide the air was evacuated from the plasma vessel with a vacuum pump until the base pressure was about 3 Pa. An appropriate gas was introduced into the vessel and the pressure was adjusted according to the desired plasma treatment. The plasma treatment involved two steps using two different gases for two samples, while the third sample was treated in one step using only oxygen. The hydrogen used in step 1 reduces oxides and the unwanted chemical components on the surface and thus increases the efficiency of step 2. The exact plasma parameters with a sample label are shown in Table 1. Two fundamentally different kinds of plasma were used: the first was H-mode plasma, where lower pressure and high power were used, while the second was E-mode, where higher pressure and lower power were used (Table 1).

*X-ray photoelectron spectroscopy (XPS)*. The XPS analyses of the samples were carried out with a PHI-TFA XPS spectrometer produced by Physical Electronics Inc. (Physical Electronics Inc., Chanhassen, MN, USA) and equipped with an X-ray monochromatic Al source. The analysed area was 0.4 mm in diameter, and the analysed depth was 3–5 nm. The high-energy-resolution spectra were acquired with an energy analyser operating at a resolution of about 0.6 eV measured at the Ag 3d_5/2_ peak and at a pass energy of 29 eV. During data processing, the spectra from the surface were aligned by setting the C 1s peak at 285.0 eV, characteristic for C–C bonds. The accuracy of the binding energies was about ±0.3 eV. Three different XPS measurements were performed on each sample and the average composition was calculated. The XPS depth profile analyses were performed to reveal the thickness and depth distribution of the elements in the subsurface region. We employed Ar-ion sputtering at 1 keV during the depth profiling, resulting in a sputter rate of 1.5 nm/min. The thickness of the oxide layer was estimated from the elemental curve for oxygen in the XPS depth profiles. The position of the oxide/matrix interface was evaluated at the point where the concentration of oxygen was reduced to half of its maximum value. The sputtering time needed to reach this interface was converted to depth using the known sputtering rate of 1.0 nm/min.

*Atomic force microscopy (AFM)*. Changes in the surface morphology of the samples were analysed using an atomic force microscope (AFM) Solver PRO (NT-MDT, Moscow, Russia) in non-contact mode in the air. Samples were placed onto standard AFM holders, and the surface was scanned using a standard Si cantilever with a force constant of 22 Nm^−1^ and at a resonance frequency of 325 kHz. The cantilever’s tip radius was 10 nm, the tip length was 95 µm, and the scan rate was set at 1.2 Hz. Every measurement was repeated at least five times. The Nova AFM software (NT-MDT, Moscow, Russia) was used for image processing.

*Wettability*. A surface-energy-evaluation system (Advex Instruments s.r.o.) was used to measure the static water-contact angles at room temperature and ambient humidity. Water droplets of 5 μL were placed on four random spots across the surface to avoid the influence of roughness and gravity on the shape of the droplet. A Young–Laplace fitting was employed to evaluate the contact angles.

*Potentiodynamic measurements*. The potentiodynamic measurements were performed in simulated physiological Hank’s solution (8 g/L NaCl, 0.40 g/L KCl, 0.35 g/L NaHCO_3_, 0.25 g/L NaH_2_PO_4_·2H_2_O, 0.06 g/L Na_2_HPO_4_·2H_2_O, 0.19 g/L CaCl_2_·2H_2_O, 0.41 g/L MgCl_2_·6H_2_O, 0.06 g/L MgSO_4_·7H_2_O, 1 g/L glucose, all Merck chemicals) at pH = 7.8 and room temperature. The potentiodynamic curves were recorded at a 1 mV/s scan rate by using BioLogic SP-300 Model Potentiostat/Galvanostat/FRA and EC-Lab V11.27 software after 1 h of stabilisation at the open-circuit potential. The electrochemical three-electrode system was used with the test specimen embedded as a working electrode, a saturated calomel electrode as a reference electrode and a platinum mesh as a counter electrode. The measurements were repeated three times to obtain statistically relevant results.

*Cell culture and chemicals*. The cell culture media and all the chemicals used in the cell experiments were purchased from Sigma–Aldrich (Steinheim, Germany) unless otherwise stated. To assess the adhesion behaviour and cell viability of the investigated samples, we used human bone osteosarcoma cells (MG-63; ATCC^®^ CRL-1427™) that were cultured in Dulbecco’s modified Eagle’s medium (DMEM), supplemented with 4-mM l-glutamine, 10% (v/v) foetal bovine serum (FBS) and 1% Penicillin-Streptomycin under controlled conditions (37 °C, 5% CO_2_, high humidity).

*Cell adhesion to metal disks*. All the investigated samples were UV-sterilized and aseptically transferred into 24-well plates. The MG-63 cells were seeded in each well (2 × 10^4^ cells per cm^2^). After a 24-h incubation under controlled conditions, the samples with adhered cells were used for the evaluation of the cell density and viability, and for the SEM analysis.

*Cell density and viability assay*. Cells grown on the samples for 24 h were rinsed with PBS and stained with 2 μg/mL Hoechst 33342 and 2 μg/mL Propidium iodide. Hoechst 33342 stains the nuclei of all the cells blue, whereas Propidium iodide stains only the nuclei of non-viable cells (cells with damaged plasmalemma) red. Stained cells were observed with a fluorescence microscope (Axio Imager.Z1; Carl Zeiss, Jena, Germany). At least 10 images per sample for each surface type were taken randomly at 100× magnification, and each of the samples was examined in triplicate. Quantitative image analysis of the cell density and cell viability was performed using ImageJ software. All results were normalized to the surface area of the sample. Data from the cell-adhesion assay were expressed as arithmetic mean + standard deviation (SD) and statistically analysed using GraphPad Prism software (GraphPad Software, San Diego, CA, USA) using a one-way ANOVA with Bonferroni’s post-hoc test for multiple comparisons. A *p* value lower than 0.05 was considered as statistically significant. The Mg samples with attached MG-63 cells were fixated and dehydrated as described in [28].

*Scanning electron microscopy (SEM)*. The surface morphology of the untreated and plasma-treated surfaces as well as the distribution and morphology of the attached M63 cells were analysed using a field-emission SEM JEOL JSM-6500F. Prior to SEM imaging, the samples were sputtered with a 3-nm thick gold layer using a Precision Etching and Coating System (Gatan 682).

## 3. Results and Discussion

### 3.1. Surface Characterisation of Plasma-Modified Magnesium

#### 3.1.1. X-ray Photoelectron Spectroscopy

**Figure 2:**
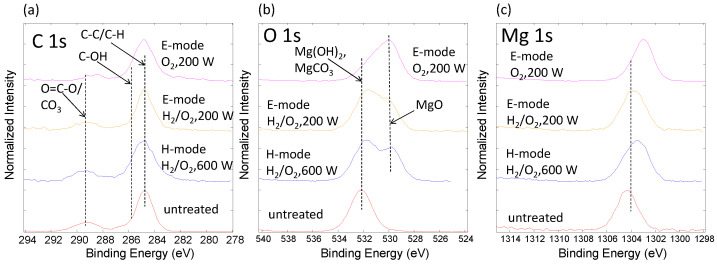
XPS spectra C 1s (**a**), O 1s (**b**) and Mg 1s (**c**) from untreated and treated magnesium samples.

The XPS analysis was applied to determine the surface composition, oxidation state of the elements and thickness of the surface oxide layer on the Mg samples after different plasma treatments. Three main chemical elements were present on the surface: oxygen, carbon, and magnesium. The chemical composition, ratio C/O and O/Mg are presented in Table 2. Figure 2 shows high-energy-resolution XPS spectra of the C 1s, O 1s and Mg 1s lines. The chemical composition changed drastically after the plasma treatment for all the treatment parameters reported herein. The increase in oxygen content was visible for the H-mode (600 W) treatment but was more pronounced for the case of both E-mode (200 W) treatments.

The hydrogen (H_2_) plasma treatment in Step 1 was designed to reduce the native magnesium oxides and carbon impurities to prepare a pristine foundation for Step 2. The C/O ratio decreased drastically after the plasma treatment due to both the removed carbon and the newly formed magnesium oxide. As can be observed, similar results can be achieved for the E-mode plasma treatment without Step 1, the most notable difference being the O/Mg ratio.

The high-energy-resolution XPS spectra shown in Figure 2 offer some additional information about the oxidation state of the elements. We found that carbon was present on the surfaces of all samples; it mainly originates from carbon-based species related with C-C/C-H bonds, C-OH bonds, and CO_3_ bonds (Figure 2a). We should note that a peak at 289.2 eV in the C 1s spectra had a FWHM of about 1.9 eV, so it was impossible to differentiate between the O=C-O and CO_3_ groups. We suppose that both of these groups may co-exist on the Mg surface. Oxygen on the untreated sample was mostly bonded to carbon C-O or Mg(OH)_2_, MgCO_3_ (Figure 2b) [29]. After the plasma treatments, oxygen was present in the form of magnesium oxide (MgO, O^2−^) due to the peak shift in the O 1s spectra from 532.0 eV (C-O, CO_3_ and OH bonds for untreated sample) to 530.0 eV characteristic for MgO and observed for all plasma-treated samples. The Mg 1s peak also showed a small shift after plasma treatments (Figure 2c) to a lower binding energy, reflecting the MgO formation on the surface. The most pronounced shift to MgO was detected for the E-mode O_2_, 200-W samples, where also the lowest O/Mg ratio was calculated (Table 2). This further confirmed the most pronounced formation of MgO on this sample. The E-mode O_2_, 200-W sample also exhibited the lowest C/O ratio, while only a small peak corresponding to the O=C-O/CO_3_ component was observed (Figure 2a). Interestingly, the E-mode H_2_/O_2_, 200-W sample seemed to have a higher carbon content and the highest O/Mg ratio of all the plasma-treated samples. From the high-resolution spectra, it could be estimated that both the E and H-mode hydrogen and oxygen plasma-treated surfaces had similar Mg(OH)_2_ and MgO peaks. In both cases, the Mg(OH)_2_ component prevailed over the MgO.

**Figure 3:**
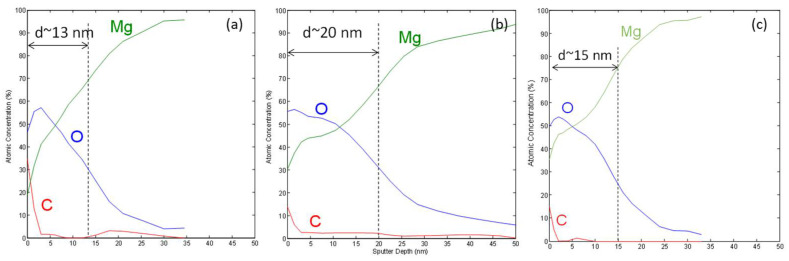
XPS depth profiles of (**a**) untreated Mg, (**b**) E-mode H_2_/O_2_, 200-W, and (**c**) H-mode H_2_/O_2_, 600-W samples.

The XPS depth profile (Figure 3) revealed the thickness of the oxide layers to be approximately (13 ± 2) nm, (15 ± 2) nm, and (20 ± 2) nm for the untreated polished, H-mode H_2_/O_2_ 600-W, and E-mode H_2_/O_2_ 200-W treated magnesium samples, respectively. The oxide thickness was not measured on the sample E-mode O_2_/200-W sample. A greater oxide thickness was obtained for all the plasma-treated samples, while a thicker oxide layer was achieved for the E-mode plasma, mainly due to the longer treatment time at lower powers (300 s at 200 W compared to 10 s at 600 W).

#### 3.1.2. Atomic Force and Scanning Electron Microscopy

**Figure 4:**
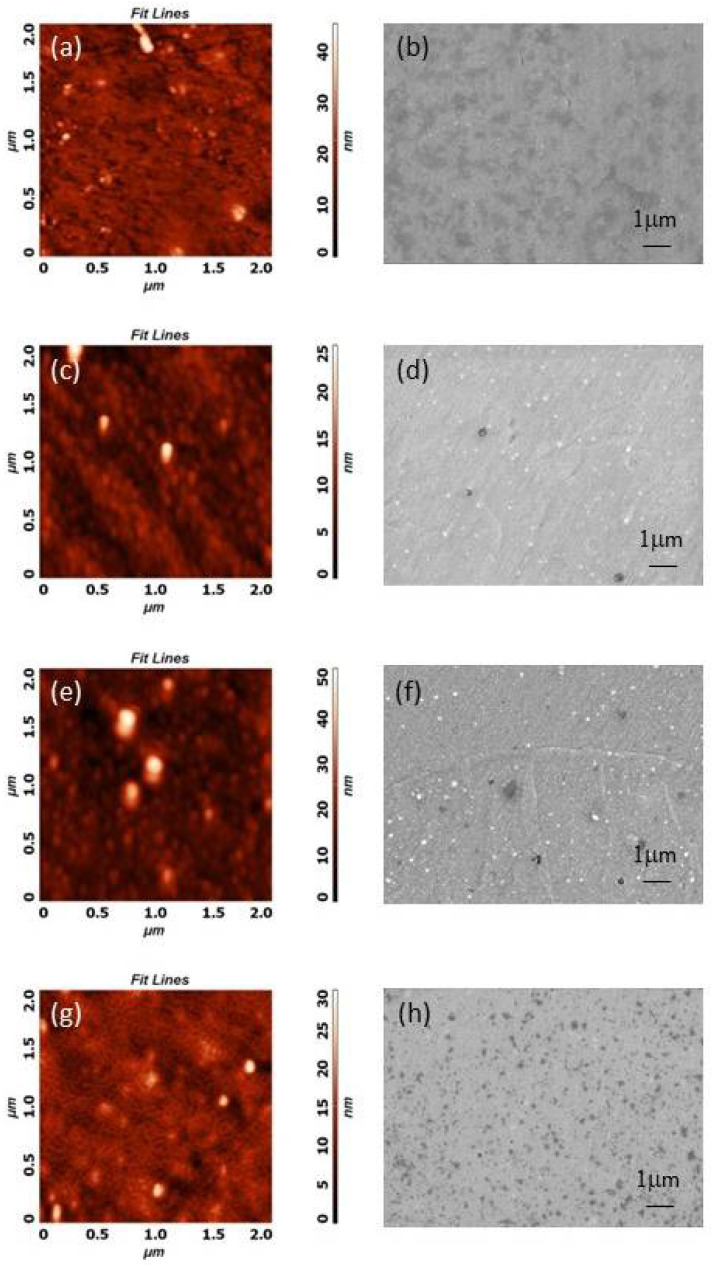
AFM images of 2 × 2 µm^2^ areas and SEM images of (**a**,**b**) untreated Mg, (**c**,**d**) E-mode O_2_, 200-W Mg, (**e**,**f**) E-mode H_2_/O_2_ 200-W and (**g**,**h**) H-mode H_2_/O_2_, 600-W Mg samples.

Fine micro-changes to the magnesium surface could be observed by both AFM and SEM because samples were polished beforehand. As can be seen from Figure 4a,b, the untreated sample exhibited a small grain-like structure with uneven distributions. Both E-mode samples were similar (Figure 4c–f), with fairly uniform grain-like structures, with more pronounced and larger features compared to the untreated sample. The H-mode H_2_/O_2_, 600-W sample appeared to have a different structure (Figure 4g,h). In this case, a grain-like structure seems to be diminished, most probably due to large thermal effects that were present in H-mode plasma. The plasma treatment in H mode was subjected to higher input power (600 W) which increased thermal heating mainly due to ion bombardment. The temperature in this case may rise to about 400 °C. While heating of the sample in E mode (200 W) was much lower, almost at room temperature. The grain height range was higher for the E-mode samples, which can be explained by a longer treatment time compared to the H-mode treated samples.

#### 3.1.3. Wettability

To analyse the surface wettability, we performed five static contact-angle measurements with water (W) on different spots all over the sample and used them to determine the average contact-angle values. As shown in Table 3, all the samples were hydrophilic. The contact angle of the untreated, diamond-polished Mg was the least hydrophilic, with a contact angle of 65°. The hydrophilicity of the H-mode H_2_/O_2_ 600-W sample was, however, slightly increased, which was reflected in the smaller contact angles. The most hydrophilicity was observed for both E-mode samples with contact angles of 25–29°. This trend can be explained with the XPS results, which indicate a decrease of the C content on all the plasma-treated samples compared to the untreated Mg. It is known that surface wettability is governed by the adsorption of organic matter from the atmosphere [30,31], which is observed for the larger contact angle for the untreated Mg compared to the plasma-treated samples. However, the wettability could be altered also due to the modified surface nanotopography caused by plasma, as seen from Figure 4, where both E-mode plasma-treated surfaces have a similar nanotopography and similar wettability.

### 3.2. Electrochemical Evaluation

**Figure 5:**
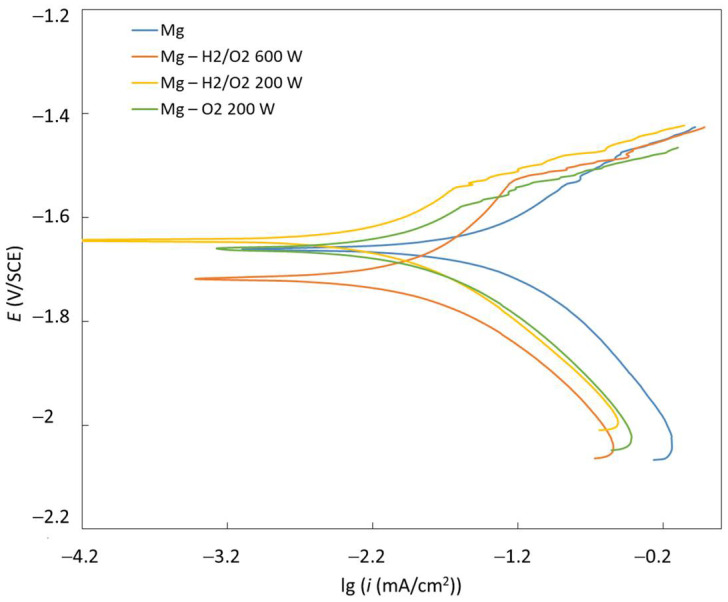
Potentiodynamic curves for untreated and plasma-modified magnesium measured in simulated physiological Hank’s solution at pH = 7.8 and room temperature.

Potentiodynamic polarisation curves of untreated Mg, H_2_/O_2_ 600-W, H_2_/O_2_ 200-W, and O_2_ 200-W samples were measured in Hank’s solution (Figure 5). Corresponding corrosion potentials (*E*_corr_), corrosion current densities (*i*_corr_), and corrosion rates (*v*_corr_) are presented in Table 4. The calculations of the *v*_corr_ and the *i*_corr_ were made according to ASTM G102-89 (2015) [32]. We observed a significant decrease of *i*_corr_ and *v*_corr_ for all the plasma-treated samples compared to the untreated Mg. The *v*_corr_ for untreated Mg was approximately 1.6 mm/year. The H-mode samples with lower pressure and higher power (H_2_/O_2_ 600 W, Mg) exhibited significantly lower *v*_corr_, both around 0.6 mm/year. The highest corrosion resistance was observed for both E-mode samples with higher pressure and lower power (H_2_/O_2_ 200 W and O_2_ 200 W) having *v*_corr_ 0.2 and 0.3 mm/year, respectively. According to the trend in contact angles, as listed in Table 3, we would intuitively expect a decreased corrosion resistance for the plasma-treated samples compared to the untreated Mg. However, the increased corrosion stability of plasma-treated Mg can be attributed to the increased oxygen content on the surface, as determined by XPS as well as the formation of a MgO component, which was detected on all the plasma-treated surfaces (Figure 2). The highest content of MgO was detected on the E-mode O_2_, 200-W sample, where the corrosion resistance was 0.3 mm/year. The highest oxygen concentration was measured for the E-mode H_2_/O_2_, 200-W sample, where the highest O/Mg ratio was also observed. This type of sample actually exhibited the most optimal corrosion resistance (0.2 mm/year).

### 3.3. Biocompatibility Evaluation

**Figure 6:**
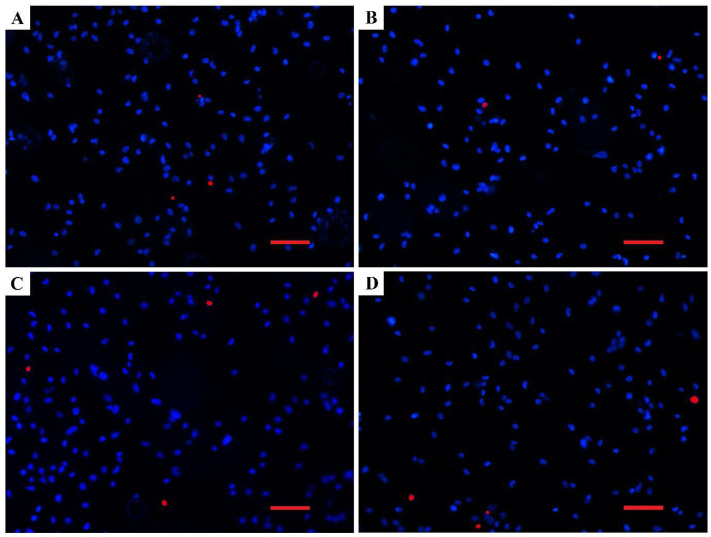
Representative fluorescence images of MG-63 cells grown on (**A**) Mg, (**B**) E-mode O_2_, 200 W, (**C**) E-mode H_2_/O_2_, 200 W and (**D**) H-mode H_2_/O_2_, 600 W for 24 h. Blue fluorescence represents the cell nuclei of viable cells and red fluorescence represents the cell nuclei of cells with damaged plasmalemma. Scale bar = 100 µm.

**Figure 7:**
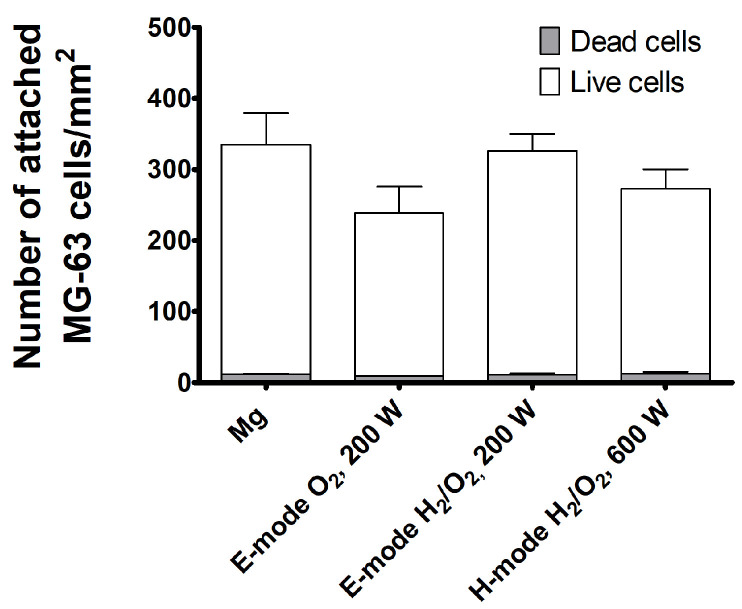
Adhesion of MG-63 cells to untreated and plasma-modified magnesium after 24 h incubation.

**Figure 8:**
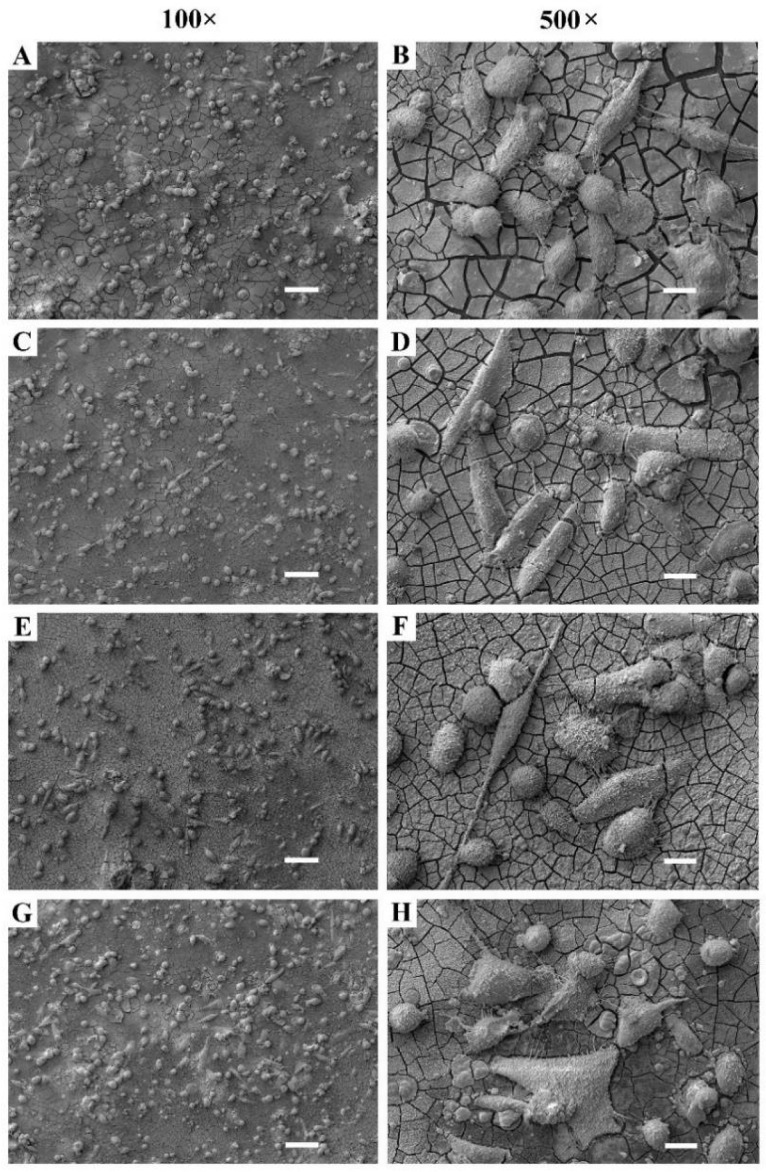
SEM images of MG-63 cell distribution and shapes on (**A**,**B**) Mg, (**C**,**D**) E-mode O_2_, 200 W, (**E**,**F**) E-mode H_2_/O_2_, 200 W and (**G**,**H**) H-mode H_2_/O_2_, 600 W. Scale bar on the 100× images is 100 µm and on the 500× images it is 20 µm.

Biocompatibility analyses revealed that the density and viability of the attached MG-63 cells (Figure 6 and Figure 7) as well as their shape (Figure 8) were not changed significantly by the different modifications of the Mg samples. The number of attached live cells is the highest for the untreated and E-mode H_2_/O_2_ 200-W samples. Despite the slight variation in attached live cells among the samples, the number of attached dead cells was very low and comparable for all the investigated samples. This confirms that plasma surface modifications did not affect the biocompatibility performance of Mg.

**Figure 9:**
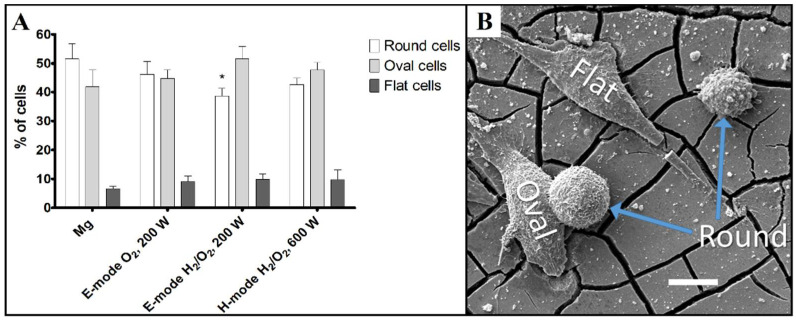
(**A**) Relative distribution of different morphological cell types of MG-63 cells attached to the samples. Data are presented as the mean percentage of all cells (+SD). Asterisk indicates significant difference in comparison to unmodified Mg sample (* *p* < 0.05; Student’s *t*-test). (**B**) SEM image showing different morphological cell types of MG-63 cells. Scale bar = 10 µm.

The SEM analysis of the samples with adherent MG-63 cells revealed that the cell morphology was not significantly changed by the Mg surface modifications. Most cells had a round or oval shape and up to 10% of cells had a flat morphology. We observed slightly more round cells on the untreated Mg sample compared to the modified samples (Figure 9). This could be due to better corrosion resistance of the modified surfaces and better cell adhesion to the plasma-modified surfaces, which was already shown to improve the proliferation of osteoblast cells [33].

## 4. Conclusions

Different plasma surface modifications of magnesium were studied to tailor the surface properties to achieve improved corrosion and biocompatibility. The samples were treated in two steps. In step 1, hydrogen plasma was used to reduce the native magnesium oxide layer. In step 2, oxygen plasma was used to enable the formation of an improved oxide layer, either by H-mode with lower pressure and high power (H-mode H_2_/O_2_, 600W) or by E-mode with higher pressure and lower power (E-mode H_2_/O_2_, 200W). In one case, only the oxygen plasma was tested to observe whether it can improve the native oxide layer, without the need to use hydrogen. The results are summed in Table 5.

Surface composition, oxidation state of the elements, and the thickness of surface oxide layer on the Mg samples after different plasma treatments, were determined using XPS. A significant decrease in carbon-based species and the formation of a 15–20-nm-thick MgO layer was observed after the plasma treatment. This also led to increased hydrophilicity of the plasma treated samples.

The corrosion resistance was significantly improved for both E-mode samples with higher pressure and lower power (H_2_/O_2_ 200 W and O_2_ 200 W), which can be attributed to an increased oxygen content on the surface as well as the formation of the MgO component, which was detected on all the plasma-treated surfaces.

The biological evaluation indicated that the biocompatibility of the plasma-treated surfaces was retained. The density and viability of the attached MG-63 cells as well as their shape were not significantly changed due to the surface modification. The morphology of the cells indicates that most of the cells had a round or an oval shape. Overall, the plasma surface modification enables us to artificially create a new, thicker, and pinhole-free oxide layer on the surface of magnesium and therefore represents an important step in the development of novel biocompatible surfaces with improved corrosion resistance for clinical use as biodegradable implants and in cardiovascular applications.

## Figures and Tables

**Table 1:** Plasma parameters used to prepare the magnesium substrates.

	Step 1				Step 2			
Sample	Gas	Pressure (Pa)	Power (W)	Time (s)	Gas	Pressure (Pa)	Power (W)	Time (s)
E-mode O_2_, 200 W	/	/	/	/	O_2_	40	200	300
E mode H_2_/O_2_, 200 W	H_2_	25	600	10	O_2_	40	200	300
H mode H_2_/O_2_, 600 W	H_2_	25	600	10	O_2_	25	600	10

**Table 2:** Chemical composition in at% of surfaces derived from XPS analyses.

Sample	Chemical Composition (at%)				
	C	O	Mg	C/O	O/Mg
Mg	48.6	37.5	14.0	1.30	2.68
E-mode O_2_, 200 W	14.6	45.9	39.5	0.32	1.16
E mode H_2_/O_2_, 200 W	21.3	49.9	28.8	0.43	1.73
H-mode H_2_/O_2_, 600 W	23.4	44.7	31.9	0.52	1.40

**Table 3:** Water contact angles (θ^W^) for untreated and plasma-modified magnesium.

Sample	Ɵ^W^ (°)
Mg	65 ± 3
E-mode O_2_, 200 W	25 ± 1
E-mode H_2_/O_2_, 200 W	28 ± 1
H-mode H_2_/O_2_, 600 W	43 ± 2

**Table 4:** Electrochemical parameters determined from the potentiodynamic curves.

Sample	*E*_corr_ (V)	*i*_corr_ (µA/cm^2^)	*v*_corr_ (mm/year)
Mg	−1640 ± 2	35.4 ± 0.5	1.62 ± 0.05
H mode H_2_/O_2_, 600 W	−1712 ± 3	13.3 ± 0.2	0.61 ± 0.2
E-mode H_2_/O_2_, 200 W	−1634 ± 2	4.1 ± 0.1	0.18 ± 0.1
E-mode O_2_, 200 W	−1641 ± 2	6.1 ± 0.1	0.28 ± 0.1

**Table 5:** Surface properties of Mg prior and after the plasma treatment.

Evaluation	Prior the Plasma Treatment	After the Plasma Treatment
XPS	Increased C/O and O/Mg ratio	Decreased C/O and O/Mg ratio
Wettability	Hydrophilic	More hydrophilic
Corrosion	Higher *v*_corr_	Decreased *v*_corr_
Biocompatibility	good	good

## Data Availability

Not applicable.
